# Development of a coding SNP panel for tracking the origin of whole-exome sequencing samples

**DOI:** 10.1186/s12864-024-10052-4

**Published:** 2024-02-05

**Authors:** Yong Huang, Yuanyuan Xiao, Shengqiu Qu, Jiaming Xue, Lin Zhang, Li Wang, Weibo Liang

**Affiliations:** 1 West China Second University Hospital, Sichuan University, Chengdu, Sichuan 610041 P.R. China; 2 Key Laboratory of Birth Defects and Related Diseases of Women and Children (Sichuan University), Ministry of Education, Chengdu, Sichuan 610041 P.R. China; 3 Department of Forensic Genetics, West China School of Basic Medical Sciences & Forensic Medicine, Sichuan University, Chengdu, Sichuan 610041 P.R. China; 4 Department of Medical Genetics, West China Second University Hospital, Sichuan University, Chengdu, Sichuan 610041 P.R. China

**Keywords:** Coding single-nucleotide polymorphisms, Whole-exome sequencing, Sample tracking, Personal identification, Parentage test

## Abstract

Whole-exome sequencing (WES) is widely used to diagnose complex genetic diseases and rare conditions. The implementation of a robust and effective quality control system for sample identification and tracking throughout the WES process is essential. We established a multiplex panel that included 22 coding single-nucleotide polymorphism (cSNP) loci. The personal identification and paternity identification abilities of the panel were evaluated, and a preliminary validation of the practical feasibility of the panel was conducted in a clinical WES case. These results indicate that the cSNP panel could be a useful tool for sample tracking in WES.

**Supplementary Information:**

The online version contains supplementary material available at 10.1186/s12864-024-10052-4.

## Background

In recent years, rapid advances in sequencing technologies have provided new options for diagnosing rare diseases [1, 2]. The remarkable success of WES in gene identification has led to its widespread integration into clinical practice [3–5]. However, the WES testing process involves a complex series of steps, making it prone to errors such as sample-labeling errors, sample mix-ups, and cross-contamination [6–8]. If such errors occur, it could potentially result in incorrect or delayed reporting of results, consequently affecting the decisions made by clinicians and the diagnosis and treatment of patients. Furthermore, errors can compromise the reliability of research findings based on this method. Therefore, sample tracking is crucial for the correct identity and accuracy of WES results. Current clinical quality control methods rely predominantly on automated equipment and the inclusion of internal quality control DNA. Genetic approaches have demonstrated efficacy in sample tracking, with multiple researchers advocating for the establishment of a quality control system for sample identification and tracking using WES to prevent testing errors [9–12]. Trio samples should be tested in parallel with WES to enhance the precision and interpretability of the quality control system results [13, 14].

Although molecular diagnostic technologies and relevant quality management are constantly improving, there is a lack of standardized methods for personal identification of WES samples. The implementation of fully automated instrumentation and information systems, although beneficial, does not entirely resolve challenges such as sample mix-ups, contamination, and the loss or deterioration of sample labels [15]. Therefore, clinical laboratories opting for genetic methods to establish personal identification, paternity tests, and sample tracking should consider independent testing methods, although creating an effective personal identification system that is practical and efficient is challenging [16]. Given the complexity and data-intensive nature of WES, and the relatively high testing costs, conducting two independent sequencing experiments simultaneously for sample identification is logistically difficult. Consequently, another genetic method should be applied to identify samples by comparing polymorphic genetic markers results. This approach allows for sample identity verification by comparing the results with the corresponding polymorphic biomarkers in the WES data, ensuring the comprehensive tracking and quality control of WES samples. Currently, the most common biomarker used for forensic personal identification and paternity testing is short tandem repeats (STRs). However, the primary location of STRs in intronic or intergenic regions makes them less compatible with the target regions of WES [17]. Furthermore, analyzing STR data within the context of WES can be challenging because of the nature of STR sequences.

In contrast, single-nucleotide polymorphisms (SNPs) are prevalent throughout the human genome, with a substantial number located in exonic (protein coding) regions [18]. SNPs involve single-base changes, and the genotyping accuracy obtained through next-generation sequencing (NGS) is superior to that of STRs [17, 19]. Thus, the development of a multiplex system using coding SNPs (cSNPs) for personal identification and parentage testing in WES offers a superior alternative to using STR. Although previous studies have made strides in this direction, challenges remain because of the need for two independent sequencing processes for comparison, and because the large number of SNPs may pose difficulties in paternity testing within trio samples [20].

Several studies have explored multiplex systems based on cSNP markers [21, 22]. Although valuable, these systems have limitations in terms of their discrimination power and complexity, often necessitating multiple experiments. Notably, established cSNP multiplex systems may not fully meet the requirements for personal identification and paternity testing in an increasing number of WES samples. In this context, the implementation of SNaPshot technology offers a cost-effective and efficient approach for detecting multiple loci simultaneously [23].

In this study, we devised a multiplex system comprising 22 cSNPs using SNaPshot technology. Through a meticulous comparison of the genotyping results of these 22 cSNPs from the WES data with those obtained using the established system, coupled with cumulative paternity index (CPI) calculations, we achieved initial sample identity alignment and confirmed parentage relationships within trio samples.

## Methods

### Sample collection and extraction

Blood samples were collected from 114 unrelated individuals, 12 parent–child pairs, and 9 trios (one of which had WES data) with their informed consent. DNA was extracted from the samples using the phenol–chloroform method [24], followed by quantification using a NanoDrop 1000 Spectrophotometer (Thermo Fisher Scientific, Wilmington, DE, USA). The DNA extracts were stored at –20 °C.

### cSNP selection and primer design

As the target region for WES is the exon region, we focused on screening candidate loci in this area. VCFtools [25] and BCFtools [26] were used to filter SNPs from the exon sequence data downloaded from the ExAC database (version r1). SNPs were further filtered by the following criteria: (1) SNPs minor allele frequency (MAF) ≥ 0.3 or allele amounts ≥ 3 were initially screened out in order to keep as many highly polymorphic SNP loci as possible; and (2) synonymous (silent) SNPs should be selected to avoid SNPs associated with diseases. Based on the primary candidate loci, SNPs were further optimized according to the following criteria: (1) biallelic SNPs with MAF ≥ 0.4 or triallelic SNPs with MAF ≥ 0.1; (2) SNPs with high specificity after screening the obtained ± 400 bp sequences of cSNPs to assess their specificity; (3) no SNPs in the selected cSNP ± 400 bp sequences in the genome; (4) the top six loci with the highest MAF on each autosomal chromosome and multi-alleles (more than two alleles) were selected; (5) the distance between two SNPs in the same chromosome is greater than 10 Mb.

For the selected cSNPs, amplification and sequencing primers were designed using Primer 3 online (http://primer3.ut.ee/) and SBEprimer software. All primers were synthesized by Thermo Fisher Scientific (Waltham, MA, USA) and verified for specificity by confirming their binding and dimerization using the AutoDimer software. Finally, SNPs with high polymorphisms and primer specificity were prioritized for inclusion in the system.

### Multiplex amplification

In this study, we used SNaPshot, a common SNP detection technology, to establish a multiplex panel. The first step of the amplification reaction included 5 μL of 2 × Taq reaction mix (QIAGEN, Hilden, Germany), 2 μL of RNase-free water, 2 μL of primer mix, and 1 μL of DNA. The reaction conditions consisted of pre-denaturation at 95 °C for 15 min, followed by 30 cycles of 94 °C for 30 s, 58.5 °C for 90 s, 72 °C for 60 s, with a final extension at 60 °C for 30 min and then holding at 4 °C. The first amplification products were digested with 1 µL of shrimp alkaline phosphatase (SAP, 1U) (New England Biolabs, USA) and 0.6 µL of exonuclease I (EXO I, 4U) (New England Biolabs) to remove any dNTPs and primer sequences. The samples were incubated for 1 h at 37 °C, followed by 12 min at 80 °C. Subsequently, the purified products underwent single-base extension (SBE) reaction with a reaction volume of 5 µL, including 1.5 μL SNaPshot ready reaction mix (Applied Biosystems, Warrington, UK), 1.3 μL extension primers mix, 1.5 μL enzyme-treated PCR product, and 0.7 μL nuclease-free water. The reaction conditions for SBE consisted of 95 °C for 10 s, 53 °C for 5 s, and 60 °C for 30 s for 25 cycles, with a hold at 4 °C. The products were further purified by adding 1 μL SAP enzyme to remove any remaining dNTPs, incubated for 1 h at 37 °C, followed by 12 min at 80 °C. All reactions were performed using an Eppendorf 6331 Nexus Gradient Flexlid Thermal Cycler (Eppendorf, Hamburg, Germany).

### Sensitivity study

To assess the sensitivity of the multiplex panel, we used varying amounts (10, 8, 6, 4, 2, 1, 0.5, and 0.1 ng) of DNA input. Each sample was subjected to the same conditions, with the only difference being the amount of DNA input.

### Capillary electrophoresis and statistical analysis

The amplification products were analyzed using a 3130 Genetic Analyzer (Applied Biosystems). 1 µL product was added in a 9 µL mixture of Hi-Di formamide (Applied Biosystems) and Liz 120 (Applied Biosystems) internal standard. The resulting mixture was thoroughly mixed, and the injection voltage and time were set to 3kv and 10 s, respectively. The raw data were analyzed using GeneMapper software version 3.2 (Applied Biosystems) with a threshold value of 50 RFU. Exact tests for Hardy–Weinberg Equilibrium (HWE) and linkage disequilibrium were calculated using Arlequin statistical software version 3.5 [27]. *P*-values < 0.05 were indicative of deviation from the HWE. We used PowerStats v1.2 (Promega, Madison, WI, USA) to calculate the allele frequency, match probability (MP), discrimination power (DP), power of exclusion (PE), and paternity index (PI).

### Extraction of cSNP genotyping from WES data

We used Basic Local Alignment Search Tool (BLAST) to select sequences that specifically represented each cSNP locus. Based on these sequences and a specific algorithm, we extracted the corresponding allele information from the trio's WES data. Subsequently, alleles with low read counts were filtered out to obtain the genotype of each locus in the sample, and the respective allele read counts. We used likelihood ratio (LR) method [28] to evaluate the identity of the samples. The LR is defined as the ratio of two conditional probabilities: 1) Hypothesis of prosecution (Hp): the probability that the genotype combination of the examinee matches that of the person of interest; and 2) Hypothesis of defense (Hd): the probability that the genotype combination of an unrelated random individual matches that of the person of interest.

## Results

### Marker selection and general information

A total of 23,782 SNPs located in exonic regions with MAF ≥ 0.3 were initially identified, of which 4,403 SNPs had allele counts ≥ 3. Subsequently, the top six cSNPs with the highest MAF on each chromosome were selected to yield a candidate database of 108 cSNPs. Further filtering based on one locus per chromosome resulted in the selection of 22 cSNPs for the multiplex panel. Details regarding the physical locations, mutation types, primer sequences, and other relevant information are summarized in Table 1. All markers were validated using polyacrylamide gel electrophoresis (PAGE) and Sanger sequencing.

**Table 1:** PCR primers used for amplification of the cSNP multiplex reaction

dbSNP rsID	Chrome	Position^a^	Gene	Reference Alleles	Annotation	Primers	Concentrations (µM)	Product size (bp)
rs12221474	10	99,332,488	ANKRD2	A/C	synonymous	F^b^: GAGGTGAAGGTGACAGGTGG	1.4	220
						R^c^: TCATTCTCCTCCTCCTGTGC		
						S^d^: ct(gact)2CAGAGCACCCACCCC	1.6	26
rs5960	13	113,801,737	F10	C/T	synonymous	F: GAAGAGGACAGCTTGGCATG	0.3	211
						R: CTTGGTAGAGACAGTGGGCT		
						S: act(gact)2GAGGGTTTCTGTGGTGGAAC	1.2	32
rs6061243	20	61,040,453	GATA5	C/G	synonymous	F: ATCTGACTTGGCGGAGGAAG	1.6	227
						R: TGACCCCTCTGTAAACACCC		
						S: t(gact)5GCTGGGCTTGGCTTT	4.8	37
rs1128925	19	2,767,192	SGTA	G/T	synonymous	F: CGAGGTGTCTGTGGGGATG	0.5	162
						R: TTGGGAGGAGAGGACAGCG		
						S: ct(gact)6AGGACCTGAGGAGCCC	1.6	43
rs9620123	22	22:43,614,316	SCUBE1	C/G	synonymous	F: CTGCATCTCTCTGTCCCCTC	2.4	166
						R: TATGTCCAGGTCTCAGGCAC		
						S: act(gact)7AGAGGCCAGCCAAGGC	5.2	48
rs8048410	16	1,614,097	IFT140	A/G	synonymous	F: TACAACAGGCAGAGCGTACC	2.4	279
						R: AATGTGTGTGGGAGGGAGAC		
						S: act(gact)8AAACAGCCGGGGCTC	6	51
rs6503070	17	7,948,175	ALOX15B	C/T	synonymous	F: CATTTGAGTGACCCCGTTCC	3	156
						R: GTGACGGGGAAGTTCTTTGG		
						S: act(gact)8GAGCACTGGCAGGAGGA	5.2	53
rs231399	4	2,831,383	SH3BP2	T/G	synonymous	F: GCCCACTCCTTTACCTCCAA	3.8	235
						R: CACTCTCCCGGAAGCAAGG		
						S: act(gact)9CCTCTTGGAGTCCTCAGC	5.6	58
rs12990557	2	202,342,402	STRADB	G/T	synonymous	F: TCTGGTGATGGCCTAGTGAC	0.14	175
						R: CAGACAACCCAATCCAACGG		
						S: ct(gact)10ACCAAACTATGCAGATGGGA	1.4	63
rs2297079	9	421,032	DOCK8	C/G	synonymous	F: CAGTACCCAAGTCCTGCAGA	2	254
						R: ACACAGACTCCCAGAATCCG		
						S: t(gact)12GCTGGAAGAGGCTTTGCT	3.8	68
rs12179	7	150,557,622	AOC1	G/A	synonymous	F: GTGTCTCTGTGCATTTGGGG	2.2	292
						R: GGGTCGTTCTGGTGGTAGAT		
						S: t(gact)13TGACCAAGTACCGGGAGTC	5.2	73
rs3734557	6	40,360,465	LRFN2	A/G	synonymous	F: GATGACGATGAAGACCAGCA	2.2	274
						R: TCAGGGACTGGCTACGACTT		
						S: t(gact)15CATGGTGCCGCCCAG	6	77
rs1051614	1	154,744,807	KCNN3	C/G	synonymous	F: GTGCCGTCCAGAAGAACTTG	2	278
						R: CAAGGCCCCTAAAGAGCATG		
						S: (gact)15AGCATCTCCAGGCTGATGTA	3.2	81
rs2279819	3	125,726,048	SLC41A3	G/C	synonymous	F: AGTGGTTGTCAGGATCCAGG	1.5	224
						R: TCAGACCCCAAGAGAAGCTG		
						S: (gact)16GTGACAATCCTGCTGTACCT	3.2	85
rs2304035	5	168,176,517	SLIT3	A/G	synonymous	F: GTGCATCTTCGCCATCTTCC	3	231
						R: AGAAACCTCCTGTCCAACCC		
						S: (gact)17CCATTTTTCCTCAAGGAGAT	8	89
rs6559167	8	6,389,889	ANGPT2	C/A/G	synonymous	F: CACCGTGTGCTTTATGTGGC	1.3	266
						R: AGGGAGGAGACGACAAACAT		
						S: (gact)18AACCTGTTGAACCAAACAGC	1.6	93
rs3741097	11	134,244,123	GLB1L2	C/G	synonymous	F: AATTCCCAGCATCCTACCGT	2	274
						R: ACCAACCTCCAGCTTCAGAA		
						S: (gact)19TTCAGAAAGGTGTCACAAGG	2.4	97
rs4758686	12	122,623,000	MLXIP	T/C	synonymous	F: AGACCAGTCACGCCATCAC	2.2	144
						R: GCCCAGAAGCTCACATGATG		
						S: (gact)20GCCATCACACTGCAGAAGAC	2.8	101
rs3737171	14	57,052,511	TMEM260	G/T	synonymous	F: GCTGCTTGAGAAAAGGGCTA	1.8	197
						R: CACAGAGAAGATTGACGCGG		
						S: (gact)21ATTGCCAGTTTAGCCACCAG	3	105
rs3744877	18	77,894,844	ADNP2	G/A	synonymous	F: GCAACTTCTGGGGTTCTTCC	2.4	185
						R: ACAACAGCTGAGGAGGAGAC		
						S: (gact)22TTGGGAGAAAGAAGCCCAGA	7.2	109
rs2249057	21	47,773,103	PCNT	C/A	synonymous	F: ACTCCGTTATGTTGCAGAGC	3	247
						R: GGAACTGCTTTGCTTACCCA		
						S: (gact)23ATCCTCAGTTGCTCCAGTTC	4	113
rs3743399	15	89,398,330	ACAN	G/A	synonymous	F: CGCAGCAACAGAGGAAAGTA	3	170
						R: CCCCAGATTCCTCCCCAGA		
						S: act(gact)23GAAGGTGTATACGGCTCTTC	4	116

^a^Position as defined in genome reference assembly GRCh37 (hg19); ^b^Forward primer for cSNP loci; ^c^Reverse primer for cSNP loci; ^d^SBE primer for cSNP loci

### Sensitivity study

**Fig. 1:**
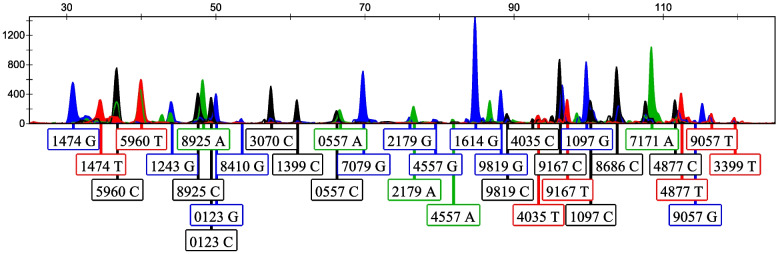
A full cSNP profile was obtained when the DNA input was 0.5 ng. X-axis—the detected genotypes of cSNP loci. Each peak corresponds to the allele of each locus, with the locus name abbreviated to the last four digits of its respective rs number

A sensitivity test was performed using random samples diluted to serial DNA amounts of 10, 8, 6, 4, 2, 1, 0.5, and 0.1 ng. Full profiles were obtained when the DNA amount > 0.5 ng (Fig. 1). When the template decreased to 0.1 ng, a partial profile was obtained with 14 cSNPs, and the highest peak height was 283 RFU. The profiles of the other DNA amounts are shown in Additional file 1: Figure S1.

### Statistical analysis

Using our multiplex panel of 22 cSNPs, we genotyped 114 unrelated individuals. The amplicon sizes ranged from 26 to 116 bp. Forensic parameters, including DP, PE, MP, polymorphism information content (PIC), and typical paternity index (TPI) were computed for the 22 cSNPs (Table 2). The cumulative matching probability of the panel was 3.314 × 10^–10^. Following the Bonferroni correction, a *P*-value threshold of 0.000216 was applied, with no deviations from the linkage equilibrium between pairwise loci. The average DP and PE values were 0.627 (range: 0.549–0.765) and 0.179 (range: 0.139–0.229), respectively. The combined DP and PE values were 0.99997 and 0.98726, respectively. Additionally, the observed and theoretical allele frequencies of the 22 cSNPs of the East Asia population are summarized in Table 3. The theoretical allele frequencies, DP, and PE of the 22 cSNPs in other populations are provided in Additional file 2: Table S1.

**Table 2:** Forensic parameters of 22 cSNPs

dbSNP rsID	DP^a^	PE^b^	MP^c^	PIC^d^	TPI^e^	HWE ^f^(*p*)
rs12221474	0.607	0.212	0.393	0.370	1.060	0.576
rs5960	0.608	0.212	0.392	0.370	1.060	0.578
rs6061243	0.625	0.173	0.375	0.370	0.970	0.850
rs1128925	0.615	0.203	0.385	0.370	1.040	0.851
rs9620123	0.609	0.212	0.391	0.370	1.060	0.707
rs8048410	0.649	0.122	0.351	0.370	0.850	0.117
rs6503070	0.601	0.188	0.399	0.360	1.000	0.694
rs231399	0.640	0.159	0.360	0.370	0.930	0.457
rs12990557	0.600	0.229	0.400	0.370	1.100	0.452
rs2297079	0.624	0.188	0.376	0.370	1.000	1.000
rs12179	0.640	0.152	0.360	0.370	0.920	0.446
rs3734557	0.631	0.162	0.369	0.370	0.940	0.700
rs1051614	0.638	0.139	0.362	0.370	0.890	0.130
rs2279819	0.647	0.145	0.353	0.370	0.900	0.266
rs2304035	0.601	0.220	0.399	0.370	1.080	0.454
rs6559167	0.765	0.169	0.235	0.510	0.960	0.001
rs3741097	0.637	0.165	0.363	0.370	0.950	0.576
rs4758686	0.643	0.139	0.357	0.370	0.890	0.256
rs3737171	0.625	0.188	0.375	0.370	1.000	1.000
rs3744877	0.638	0.162	0.362	0.370	0.940	0.572
rs2249057	0.549	0.212	0.451	0.340	1.060	0.053
rs3743399	0.602	0.195	0.398	0.370	1.020	0.696

^a^*DP* Discrimination power; ^b^*PE *Probability of exclusion; ^c^*MP *Match probability; ^d^*PIC *Polymorphism information content; ^e^*TPI *Typical paternity index; ^f^*HWE *Hardy–Weinberg *P*-value. The *P*-value is greater than 0.000216, indicating that there is no linkage between the loci

**Table 3:** The allele frequency of 22 cSNPs

dbSNP rsID	Allele	Observed Frequency	Theoretical Frequency^a^
rs12221474	A/C	0.544/0.456	0.471/0.529
rs5960	C/T	0.535/0.465	0.476/0.524
rs6061243	C/G	0.566/0.434	0.539/0.461
rs1128925	G/T	0.478/0.522	0.483/0.517
rs9620123	C/G	0.474/0.526	0.473/0.527
rs8048410	A/G	0.564/0.436	0.433/0.567
rs6503070	C/T	0.610/0.390	0.591/0.409
rs231399	T/G	0.513/0.487	0.529/0.471
rs12990557	G/T	0.491/0.509	0.506/0.494
rs2297079	C/G	0.522/0.478	0.501/0.499
rs12179	G/A	0.456/0.544	0.520/0.480
rs3734557	A/G	0.438/0.562	0.472/0.528
rs1051614	C/G	0.570/0.430	0.480/0.520
rs2279819	G/C	0.487/0.513	0.497/0.503
rs2304035	A/G	0.548/0.452	0.504/0.496
rs6559167	C/A/G	0.124/0.376/0.500	0.145/0.409/0.446
rs3741097	C/G	0.482/0.518	0.491/0.509
rs4758686	T/C	0.439/0.561	0.507/0.493
rs3737171	G/T	0.513/0.487	0.527/0.473
rs3744877	G/A	0.473/0.527	0.424/0.576
rs2249057	C/A	0.675/0.325	0.616/0.384
rs3743399	G/A	0.596/0.404	0.509/0.491

^a^The theoretical allele frequencies of the 22 cSNPs in East Asia population

### Paternity test

To validate the ability to establish parentage, we conducted tests on 12 duos and 8 trios, calculating the PI and CPI. Allele frequencies were calculated using PowerStats based on the genotyping results of 114 unrelated individuals. All parent–child pairs conformed to Mendel’s laws of inheritance. No mutations or recombinations were observed in any of the cSNP markers in the 12 parent–child pairs. The CPI averaged 66.696 (range: 2.905 to 313.697) for the 12 parent–child pairs and 950.022 (range: 63.383 to 2,908.424) for the 8 trio samples, using the 22 cSNP markers. Typically, a CPI exceeding 1,000 indicates strong support for a parent–child relationship. Moreover, the CPI tends to increase when analyzing trios simultaneously, making trio analysis useful for determining parent–child relationships.

### cSNP genotyping from WES data

**Fig. 2:**
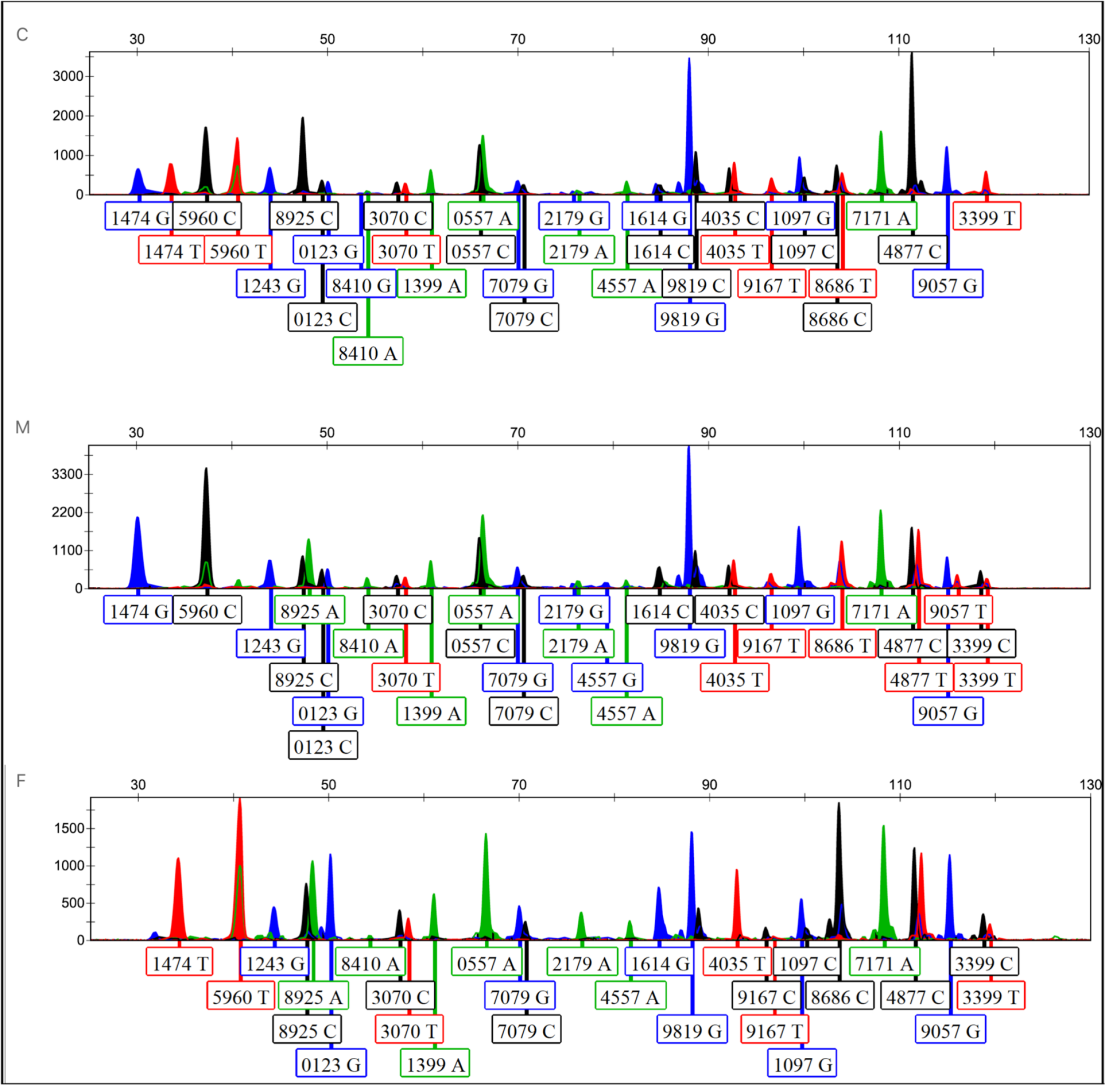
The cSNP profiles of the trio sample. C = children, M = mother, F = father

This cSNP extraction method was validated using the WES data from a trio family. The cSNP genotyping results for each extracted sample are shown in Table 4. The established cSNP system was used to analyze the trio samples in parallel. The genotyping results for each sample are shown in Fig. 2. The genotyping results obtained using the cSNP system for each sample in the trio were largely consistent with the extracted genotyping results. Finally, based on the frequency distribution of each cSNP locus in the tested population and using forensic genetic analysis methods, the CPIs for the father and mother were calculated to be 86.007, and 48.700, respectively; the MP for the child, father, and mother were calculated to be 1.289 × 10^–10^, 9.943 × 10^–11^, and 1.243 × 10^–11^, respectively; and the LRs for the child, father, and mother were calculated to be 775,526,885, 10,057,219,242, and 80,445,560,559, respectively.

**Table 4:** The extracted cSNP genotyping of WES trio samples

dbSNP rsID	C^a^				M^b^				F^c^			
rs1051614	C	13^d^	G	4	C	21			G	14		
rs12990557	C	51	A	14	C	67	A	13	C	71	A	28
rs2279819	C	21	G	14	C	16	G	10	C	14	G	15
rs231399	A	41			A	37			A	33		
rs2304035	C	16	T	7	C	12	T	12	T	25		
rs3734557	A	30			A	13	G	9	A	14		
rs12179	A	27	G	21	A	14	G	24	A	42		
rs6559167	T	51			T	45			T	17	C	17
rs2297079	G	26	C	20	G	19	C	14	G	8	C	28
rs12221474	T	18	G	17	G	25			T	35		
rs3741097	G	12	C	9	G	33			G	13	C	8
rs4758686	C	8	T	18	T	28			C	24		
rs5960	T	13	C	25	C	35			T	41		
rs3737171	A	29			A	29			A	40		
rs3743399	T	28			T	8	C	21	T	16	C	20
rs8048410	A	7	G	16	A	25			A	13	G	13
rs6503070	C	5	T	3	C	7	T	16	C	9	T	4
rs3744877	C	30			T	17	C	6	T	7	C	9
rs1128925	C	31			C	22	A	19	C	12	A	12
rs6061243	G	38			G	31			G	39		
rs2249057	G	38			T	17	G	30	G	52		
rs9620123	G	28	C	32	G	29	C	19	G	37		

^a^C = children, ^b^M = mother, ^c^F = father, ^d^The number represents the reads of each allele

## Discussion

When using WES, confirming the sample identity is crucial for obtaining reliable results. The current methods and quality control protocols in clinical laboratories fall short of fully tracking samples throughout the testing process [29]. Although the establishment of medical testing centers and adoption of new equipment have been effective in reducing the number of manual errors, challenges persist, particularly in the pretest phase [15, 30]. For example, in the case of WES samples obtained from prenatal fetal sources (such as villi, amniotic fluid, and umbilical cord blood) or from miscarriage tissues, it is only after processing the sequencing data that one can determine whether the sample is singular or mixed. However, determining whether a single sample originates from the fetus or the mother is challenging. Despite the application of fully automated equipment and information systems, that streamline manual operations, the potential for technical error persists. Addressing issues such as sample exchange, contamination, and label loss or damage remains a challenge [15]. Even when quality control materials involve exogenous DNA sequences, they only allow quality control during the post-DNA extraction detection steps [31]. This does not guarantee that the DNA sample originated from the subject of interest, so it is unable to confirm the sample identity conclusively.

The commonly used forensic STR test kits primarily include the following core loci: 1) Combined DNA Index System (CODIS), including 13 STR loci: CSF1PO, FGA, TH01, TPOX, VWA, D3S1358, D5S818, D7S820, D8S1179, D13S317, D16S539, D18S51, and D21S11, with a cumulative MP of 2.003 × 10^–15^; and 2) Expanded U.S. core loci, including the 13 CODIS loci mentioned above, and D1S1656, D2S441, D2S1338, D10S1248, D12S391, D19S433, and D22S1045, totaling 20 STR loci with a cumulative MP of 2.022 × 10^–22^ [32]. The CODIS system satisfies the basic requirements for personal identification and paternity testing. However, commercial kits available for forensic science, whether STR-based (including CODIS/expanded CODIS) or SNP-based, primarily target loci located in intronic or intergenic regions of the respective genes, rather than exons. The target region for WES is the exonic region, which cannot be directly assessed using existing forensic multiplex systems. Consequently, extracting the genotypes of these forensic loci from WES data is challenging [17]. Moreover, when samples are genotyped using methods for different target areas, comparability between the two results is compromised, rendering concordance tests unfeasible [3]. Consequently, the use of these kits for personal identification using WES, and parentage testing is limited.

The large number of SNPs in coding regions holds potential for their use in personal identification and sample tracking [33]. However, existing multiplex cSNP systems have drawbacks, including high cost, time inefficiencies, operational complexity, and inability to be applied to WES data analysis [34, 35]. Du et al. [36] developed an SNP panel comprising 74 genome-wide SNPs and introduced a user-friendly online validation tool. However, only a subset of the 74 SNPs were located in the exonic region, rendering many SNPs unsuitable for WES application. The sample consistency verification software NGSCheckMate, developed by Lee et al. [37], can be used to analyze 11,696 SNP genotypes corresponding to the same sequencing data type, the same sequencing data type but different data formats (such as FASTQ and BAM), and the same data type but different sequencing types (such as whole-genome sequencing, WES, and RNA sequencing). In addition, Wesrphal et al. [38] screened 6,000 SNPs in the human genome and devised SMaSH, a Bayesian framework adept at effectively discerning whether corresponding samples from different NGS datasets are congruent. Javed et al. [20] used approximately 60,000 SNPs based on the principle of linkage disequilibrium to establish CrosscheckFingerprints (Crosscheck), a tool capable of detecting the interchangeability of samples across diverse NGS data types. Pengelly et al. [22] identified 24 biallelic cSNPs with a theoretical cumulative MP of approximately 4.641 × 10^–10^ for CHB, which exhibited a DP significantly lower than that of CODIS, and proved inadequate for basic requirements in personal identification and paternity testing. Notably, Pengelly et al. did not establish a detection method for these cSNPs but merely suggested them as candidate biomarkers for laboratory use. Helsmoortel et al. [23] established a multiplex system containing 21 cSNPs using a high-resolution melting method with a total of eight reactions. However, this method requires multiple experimental operations and integration of results. Moreover, the DP of the 21 biallelic cSNPs falls short of the basic requirement for personal identification and paternity testing, particularly as the number of WES detection samples continues to increase. These results indicate that a method that screens for additional cSNP loci with high polymorphism is needed to construct an effective system for personal identification. Additionally, the development of software capable of analyzing the corresponding cSNP genotypes from WES data is a promising development.

In this study, we screened out 22 multi-allelic cSNPs to establish a multiplex panel for tracking WES samples. However, the observed frequency of these 22 cSNPs is lower than their theoretical frequency. This variation may be attributed to the study population. The cumulative discrimination power (CDP) and cumulative MP values are forensic parameters for evaluating the efficiency of the system for personal identification. A CDP closer to 1 suggests a higher probability that, when two unrelated individuals are randomly selected from the population, the genotyping results for the 22 cSNP will differ, signifying a robust differentiating ability based on these markers. The CDP of 22 cSNPs indicates that in a sufficiently large population, if we were to randomly select two unrelated individuals 100,000 times, the genotyping results of these 22 cSNPs will be different in more than 99,997 instances. The cumulative MP of the 22 cSNPs closer to 0 implies a reduced likelihood of a random individual matching the target individual in terms of genotyping results. These results suggest that our panel can effectively and reliably trace and identify individuals in WES sample tracing. In forensic statistics, the LR method [28] is commonly used to assess the strength of the evidence provided by genetic analysis. A higher LR value lends greater support to the prosecution's hypothesis, supporting the notion that the person of interest and the suspect are the same individual. In the WES data application, the LR values strongly support the three WES samples corresponding to the child, father, and mother.

These results signify that our panel has an extremely low likelihood of erroneously identifying samples in clinical applications. Although the MP of 22 cSNPs did not reach the level of STRs, this multiplex system provides an efficient and straightforward method for sample identity and tracking of WES samples. The multiplex panel data can be used for comparison with the corresponding cSNP genotypes in the WES data. During WES, it is possible to concurrently detect 22 cSNPs in the same sample using the capillary electrophoresis (CE) platform. On completion of the sequencing process, the genotyping results for the 22 cSNPs from both WES and the CE platform were compared to confirm the identity of the sample. This streamlined procedure saves time and is cost-effective, thereby eliminating the need for repetitive sequencing. These 22 cSNPs can be used for sample tracking throughout the WES process. Further research is needed to establish a multiplex detection system based on these 22 cSNPs and optimize the corresponding multiplex system. Additionally, more cSNP markers should be selected to improve the DP of the system. The CPI of the 22 cSNPs was lower than 10,000, because of the lower polymorphism compared with STRs. Therefore, a system that includes a greater number of multi-allelic cSNPs should be established to meet these needs. The preliminary results of the extraction method for WES data suggest that this approach can efficiently extract cSNP genotype results and perform comparisons.

In the future, we plan to develop software capable of directly, rapidly, and accurately extracting the genotyping data of all cSNP loci included in the established personal identification system, from WES data. This will enable personal identification and identity confirmation in WES samples as well as paternity tests in trio samples. Simultaneously, using the cSNP panel and WES data cSNP genotyping extraction software, we intend to develop a novel quality control framework for WES sample tracking. Ultimately, this will facilitate sample tracking throughout the WES testing process.

## Conclusions

We successfully established a multiplex panel comprising 22 cSNPs based on SNaPshot technology and applied it to personal identification and paternity testing. This multiplex system not only enhances tracking of WES samples, but also increases the overall reliability of WES detection.

### Supplementary Information

**Additional file 1.** 

**Additional file 2.** 

## Data Availability

All data generated or analyzed in this study are included in the article and supplementary material.

## Abbreviations

BLAST: Basic local alignment search tool
CDP: Cumulative discrimination power
CODIS: Combined DNA Index System
CPI: Combined paternity index
cSNP: Coding single-nucleotide polymorphism
DP: Discrimination power
EXO I: Exonuclease I
HWE: Hardy–weinberg equilibrium
LR: Likelihood ratio
MAF: Minor allele frequency
MP: Match probability
NGS: Next-generation sequencing
PAGE: Polyacrylamide gel electrophoresis
PE: Probability of exclusion
PI: Paternity index
PIC: Polymorphism information content
SAP: Shrimp alkaline phosphatase
SBE: Single-base extension
TPI: Typical paternity index
WES: Whole-exome sequencing
