# Supplementary material for: Development of a coding SNP panel for tracking the origin of whole-exome sequencing samples
Source: BMC Genomics. 2024 Feb 5;25:142. doi: 10.1186/s12864-024-10052-4 (PMC10840194; doi:10.1186/s12864-024-10052-4)
Supplement: Supplementary file 1 — Additional file 1. [file 12864_2024_10052_MOESM1_ESM.pdf]

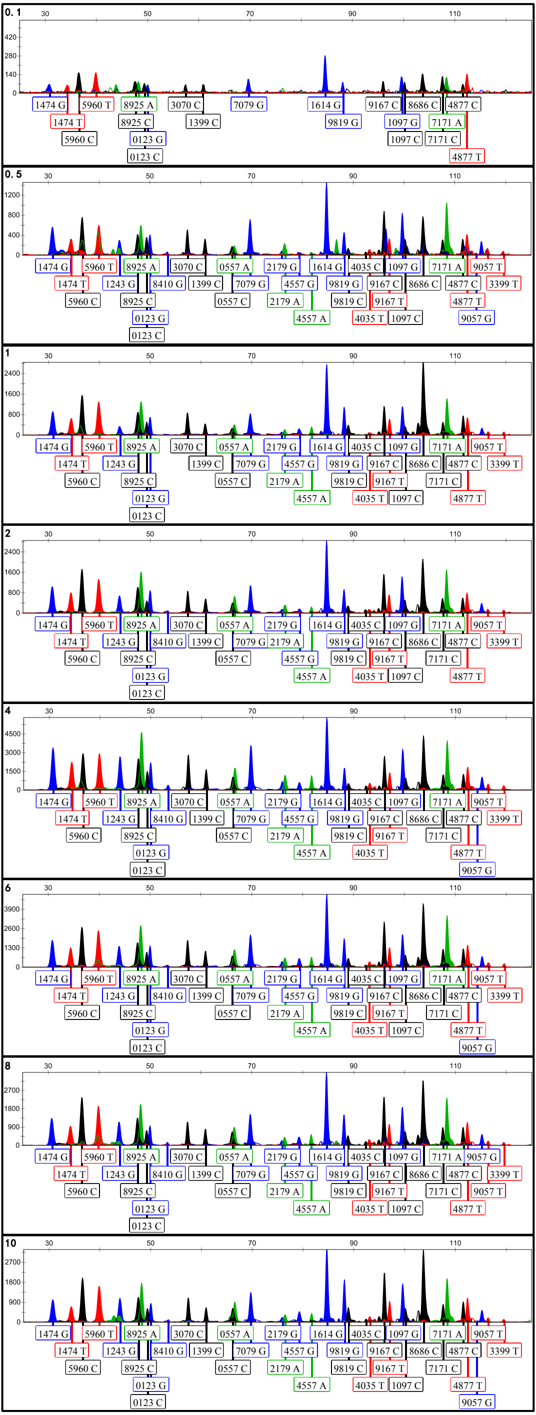


Additional file 1: The coding single-nucleotide polymorphism (cSNP) profiles for different DNA inputs. The full cSNP profiles were obtained when DNA inputs exceeded 0.5 ng, but a partial profile was obtained when DNA input was 0.1 ng.
